# Reply to the ‘Comment on “Metal–organic green dye: chemical and physical insight into a modified Zn-benzoporphyrin for dye-sensitized solar cells”’ by R. Steer, *RSC Advances*, 2018, DOI: 10.1039/c8ra00213d†

**DOI:** 10.1039/c8ra01651h

**Published:** 2018-06-04

**Authors:** G. Zanotti, N. Angelini, G. Mattioli, A. M. Paoletti, G. Pennesi, G. Rossi, D. Caschera, L. de Marco, G. Gigli

**Affiliations:** Istituto di Struttura della Materia (ISM) – CNR Via Salaria Km 29.300, 00015 Monterotondo (Rm) Italy gloria.zanotti@ism.cnr.it; Istituto per lo Studio dei Materiali Nanostrutturati (ISMN) – CNR Via Salaria Km 29.300, 00015 Monterotondo (Rm) Italy; IIT – Center for Biomolecular Nanotechnologies Via Barsanti, Arnesano (Le) 73010 – Italy; CNR-NANOTEC Via Amendola 122/D Bari 70126 Italy; Dipartimento di Matematica e Fisica, Università del Salento Lecce 73100 Italy

## Abstract

The authors reply to the comment by R. P. Steer discussing the reasons for their incorrect assignment of the luminescence decay of the novel compound 5,10,15-(triphenyl),20-[ethynyl-(4-carboxy)phenyl]tetrabenzoporphyrinate Zn(ii) (PETBP). Further DFT and TDDFT calculations have been performed on the compound to investigate the possibility of a direct S_2_–S_0_ decay instead of a S_2_–S_1_ conversion with a subsequent emission to the ground state. In addition, the presence of traces of very luminescent contaminants of the ring-opened type has been considered on the grounds of calculated absorption and fluorescence spectra. The results of these investigations confirm that the S_2_–S_0_ emission reported in the commented paper is not attributable to the target molecule but rather to a neglected luminescent impurity.

## Abstract 2

The authors reply to the comment by R. P. Steer discussing the reasons for their incorrect assignment of the luminescence decay of the novel compound 5,10,15-(triphenyl),20-[ethynyl-(4-carboxy)phenyl]tetrabenzoporphyrinate Zn(ii) (PETBP).

## Introduction

We reply to the comment written by R. P. Steer to our article “Metal–organic green dye: chemical and physical insight into a modified Zn-benzoporphyrin for dye-sensitized solar cells” published on *RSC Advances* in 2016.^1^

The comment discusses the wrong assignment of a fluorescence band to a direct S_2_–S_0_ radiative decay of our target compound, a substituted benzoporphyrin named PETBP (Scheme 1 of ref. 1), that was designed, characterized and tested as a novel green photosensitizer for hybrid-organic photovoltaics because of its appealing hybrid porphyrin–phthalocyanine structure. Our paper was focused on its synthesis and on the chemical, electrical and photophysical characterization of the free molecule and of the related DSC. The conclusion that Steer reaches, to which we fully agree, is that the fluorescence spectrum in solution that we reported cannot be attributed uniquely to our target compound, but more likely to an impurity whose emission falls in the range of wavelengths generally belonging to the S_2_–S_0_ decay of porphyrins and analogs.

**Fig. 1:**
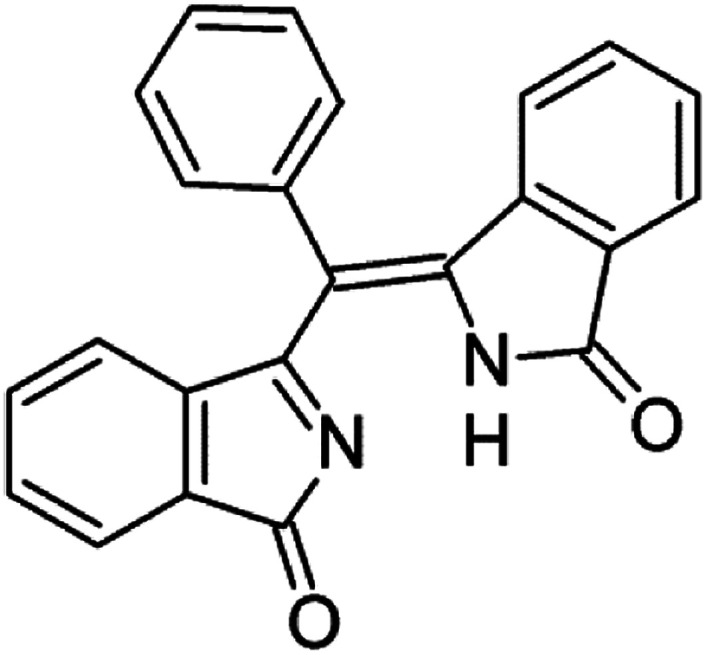
Molecular structure of (3-oxoisoindolenyl)(3 oxoisoindolinylidene)phenylmethane.

While characterizing our molecule as a luminescent chromophore and as a photosensitizer in a solar cell, we evaluated evidences that favored our mistake: the emission spectrum (Fig. 1, ref. 1) at *λ*_ex_ = 460 nm consisted of two bands, peaked around 500 and 550 nm, suggesting the possibility of a direct S_2_–S_0_ emission reported in the case of several porphyrinoid system,^2,3^ even if never characterized by such a strong intensity. Moreover, the shape of the IPCE curves (Fig. 9 of ref. 1) of our solar cells showed a significant photon to current conversion efficiency in the range of blue wavelengths (35% at 470 nm) and a less intense contribution related to the Q band at higher wavelengths (4% at 635 nm). Regarding the latter occurrence, previous results obtained in the case of a porphyrin similar to PETPB, namely YD0,^4^ and cited in our article for comparison, report an IPCE spectrum in which the conversion efficiency from B and Q bands are comparable. When confronting the relative UV-Vis absorption spectra of the two molecules, they are quite similar one another. So, we speculated that the IPCE differences could be due to a different lifetime of the S_2_ excited state of PETBP, that if long enough could have directly contributed to the charge injection into the conduction band of TiO_2_ substrate before decaying.

Moreover, we calculated an *ab initio* absorption spectrum of PETBP (Fig. 6 in ref. 1) constituted by two main transitions contributing to the Soret band, arising from the breaking of symmetry of the degenerate LUMO in PETBP with respect to the unsubstituted tetraphenyl-tetrabenzo porphyrin. This fact further gave us the impression that a direct S_2_–S_0_ emission could be plausible, because we ascribed it as a possible source of the corresponding emission spectrum broadening.

Encouraged by these evidences, and by the fulfillment of some of the requirements described by Steer in his review on the assignment of S_2_–S_0_ decays in porphyrinoid systems,^5^ we underestimated the absence of some others. The too large Stokes shift of the mid-visible emission maximum around 500–550 nm, relative to the corresponding 456 nm Soret absorption maximum, and the breaking of the “mirror image rule” should have warned us and prompted to perform further investigations, not to mention the integrated emission intensity of the erroneously assigned S_2_–S_0_ transition in the mid-visible, more than twenty times larger than the integrated intensity of the expected fluorescence of the Q band.

We agree with Steer that, while this error has slight consequences on the main goal of our paper, *i.e.* the use of PETBP as a sensitizer in DSCs, the reported data can indeed be misleading for researchers involved in the investigation of porphyrins having rather long-lived upper excited electronic states, with a potential use as dual absorber-upconverters, and it is worthwhile to clarify it completely.

## Discussion

To identify the origin of our mistake, we firstly reconsidered our experimental data. We routinely deal with the synthesis of metal–organic macrocycles, mostly phthalocyanines, with an average molecular mass around 1000 u.m.a., that we normally purify by chromatography and/or crystallization. Since any structurally-related byproduct in the crude would likely present a similar optical behavior, prior to a luminescent characterization we carefully isolate our target molecules from related macrocycles that could mislead the results. So while checking for fluorescent impurities we focused on chemical species with comparable molecular weight and spectral response and removed them when present. The data analysis is detailed in the ESI† and evidences the difficulty to determine the presence of low molecular weight contaminants, that we could not highlight with the performed characterizations. We therefore speculated about the chemical nature of the contaminant, screening several plausible structures with the assistance of *ab initio* simulations. Furthermore, we analyzed the probability to have a direct PETBP S_2_–S_0_ decay instead of a S_2_–S_1_ conversion with a subsequent emission to the ground state.

Concerning the latter, even if the probability of a S_2_–S_0_ radiative transition is stronger than the S_1_–S_0_ probability for all the investigated porphyrin systems (not reported here), we fully acknowledge the fact, thoroughly explained by Steer in his review, that a fast S_2_–S_1_ internal conversion whose rate we cannot calculate, quantitatively quenches S_2_ and does not allow a detection of the S_2_–S_0_ fluorescence in steady-state emission spectra, but as very low contributions (10^−3^ weaker in magnitude). Moreover, our TDDFT results for PETBP show two dark states between the Soret and Q band which make the probability to have a quantitative fluorescence emission from S_2_ even lower. Furthermore, we have considered the presence of traces of very luminescent contaminants and, after an initial screening of some plausible chemical structures, we have focused our attention on (3-oxoisoindolenyl)(3-oxoisoindolinylidene)phenylmethane, hereinafter named PREC, whose structure is reported in Fig. 1 and that has been synthesized by Galanin *et al.*^6^ as a direct precursor of another polisubstituted tetrabenzoporphyrin, namely meso-*trans*-diphenyldi(2-quinolyl)tetrabenzoporphine.

**Fig. 2:**
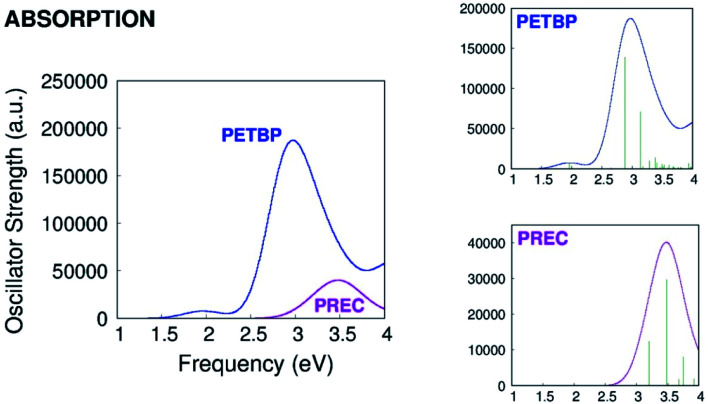
Calculated absorption spectra of PETBP and PREC.

As it can be seen in Fig. 2, the calculated absorption band of PREC is less intense than the Soret but more intense than the Q band of PETBP. The S_1_ → S_0_ quantum yield of the of the molecule, not reported by Galanin *et al.*, is probably high enough to be detected even in traces.^6^

**Fig. 3:**
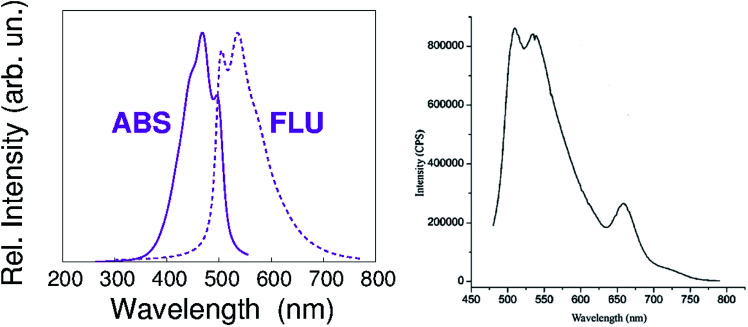
Left panel – calculated vibronic structure of the S_1_–S_0_ fluorescence of PREC. Right panel – PETBP fluorescence emission spectrum, as reported in Fig. 2 of our paper.

According to our calculations, the vibronic structure of the S_1_–S_0_ fluorescence of PREC results in the spectrum shown in Fig. 3. It is very similar to the low-wavelength contribution to the fluorescence spectrum shown in Fig. 2 of our paper, and reported here again for the sake of clarity.

Given these evidences, we agree that Fig. 2 shows the Q-band fluorescence of PETBP along with the intense response of traces of a low-molecular weight, non-macrocyclic contaminant. Its presence could be either due to an incomplete tetramerization process in the synthetic procedure that we chose^7^ or to a partial sample decomposition during the fluorescence measurements.

We acknowledge therefore the wrong assignment of Fig. 2 in our paper. However, we also note that it does not invalidate the discussion of our results related to the photovoltaic performance of PETBP: as a matter of fact, the IPCE spectrum shows a relevant charge transfer into TiO_2_ from the Soret band, even greater than that occurring from the Q band. The S_2_ lifetime in porphyrinoid systems is generally estimated around 1 ps, and then compatible with a direct electron injection from S_2_ to the TiO_2_ conduction band, which occurs within 100 fs.^8,9^

## Experimental section

### Computational details

The structural, electronic and optical properties of PETBP and PREC have been investigated using *ab initio* simulations based on density functional theory (DFT). In detail, the calculations have been performed by using the ORCA suite of programs^10^ in a localized-basis-set framework. Kohn–Sham orbitals have been expanded on a def2-TZVPP Gaussian type basis set.^11^ Fully decontracted def2-TZVPP/J has been also used as an auxiliary basis set for Coulomb fitting in a resolution-of-identity/chain-of-spheres (RIJCOSX) framework.^12^ Molecular geometries have been fully optimized at the B3LYP level of theory,^13,14^ including dispersion forces calculated by using the DFT-D3 approach^15^ TDDFT calculations have been performed by using the B3LYP functional and the same basis sets discussed above. A large basis of 500 vectors connecting occupied and unoccupied Kohn–Sham orbitals has been used for the calculations of the first 50 electronic transitions. The absorption and fluorescence vibrational structure of the spectrum has been calculated by using the independent mode displaced harmonic oscillator (IMDHO) model.^16^

## Conflicts of interest

The authors declare no conflict of interest.

## Supplementary Material

RA-008-C8RA01651H-s001
