# Supplementary material for: Reply to the ‘Comment on “Metal–organic green dye: chemical and physical insight into a modified Zn-benzoporphyrin for dye-sensitized solar cells”’ by R. Steer, RSC Advances, 2018, DOI: 10.1039/c8ra00213d
Source: RSC Adv. 2018 Jun 4;8(36):20259–62. doi: 10.1039/c8ra01651h (PMC9128345; doi:10.1039/c8ra01651h)
Supplement: RA-008-C8RA01651H-s001 [file RA-008-C8RA01651H-s001.pdf]

Electronic Supplementary Material (ESI) for RSC Advances.
This journal is © The Royal Society of Chemistry 2018

SUPPORTING INFORMATION

Reply to the 'Comment on "Metal–organic green dye: chemical and physical insight into a modified Zn-benzoporphyrin for dye-sensitized solar cells"' by R. Steer, RSC Advances, 2018, DOI: 10.1039/c8ra00213d

G. Zanotti[a], N. Angelini[a], G. Mattioli[a], A. M. Paoletti[a], G. Pennesi[a], G. Rossi[a], D. Caschera[b], L. de Marco[c] and G. Gigli[d e]

[a] Istituto di Struttura della Materia (ISM) - CNR, Via Salaria km 29.300, 00015 Monterotondo (Rm), Italy

[b] Istituto per lo Studio dei Materiali Nanostrutturati (ISMN) - CNR, Via Salaria km 29.300, 00015 Monterotondo (Rm), Italy

[c] IIT - Center for Biomolecular Nanotechnologies, Via Barsanti, Arnesano (Le), 73010 - Italy

[d] CNR-NANOTEC Via Amendola 122/D - Bari 70126-Italy

[e] Dipartimento di Matematica e Fisica, Università del Salento, Lecce, 73100 - Italy

The best way to clarify our wrong fluorescence assignment is to describe our workflow and our experimental data, which will also explain the reason for undetecting a contaminant in our product.

As reported in the main text, while checking for fluorescent impurities in the benzoporphyrin reaction mixtures we focused on chemical species with comparable molecular weight and spectral response. As an example, in figures S1a-b we report the MALDI spectrum of the crude of the non-optimized last synthetic step of **PETBP** in which at least one macrocyclic contaminant is present at m/z 877, together with our product whose diagnostic peaks are $[M+H]^+$ 949 u.m.a, $[M+Na]^+$ 967 u.m.a., $[M+K]^+$ 981 u.m.a.

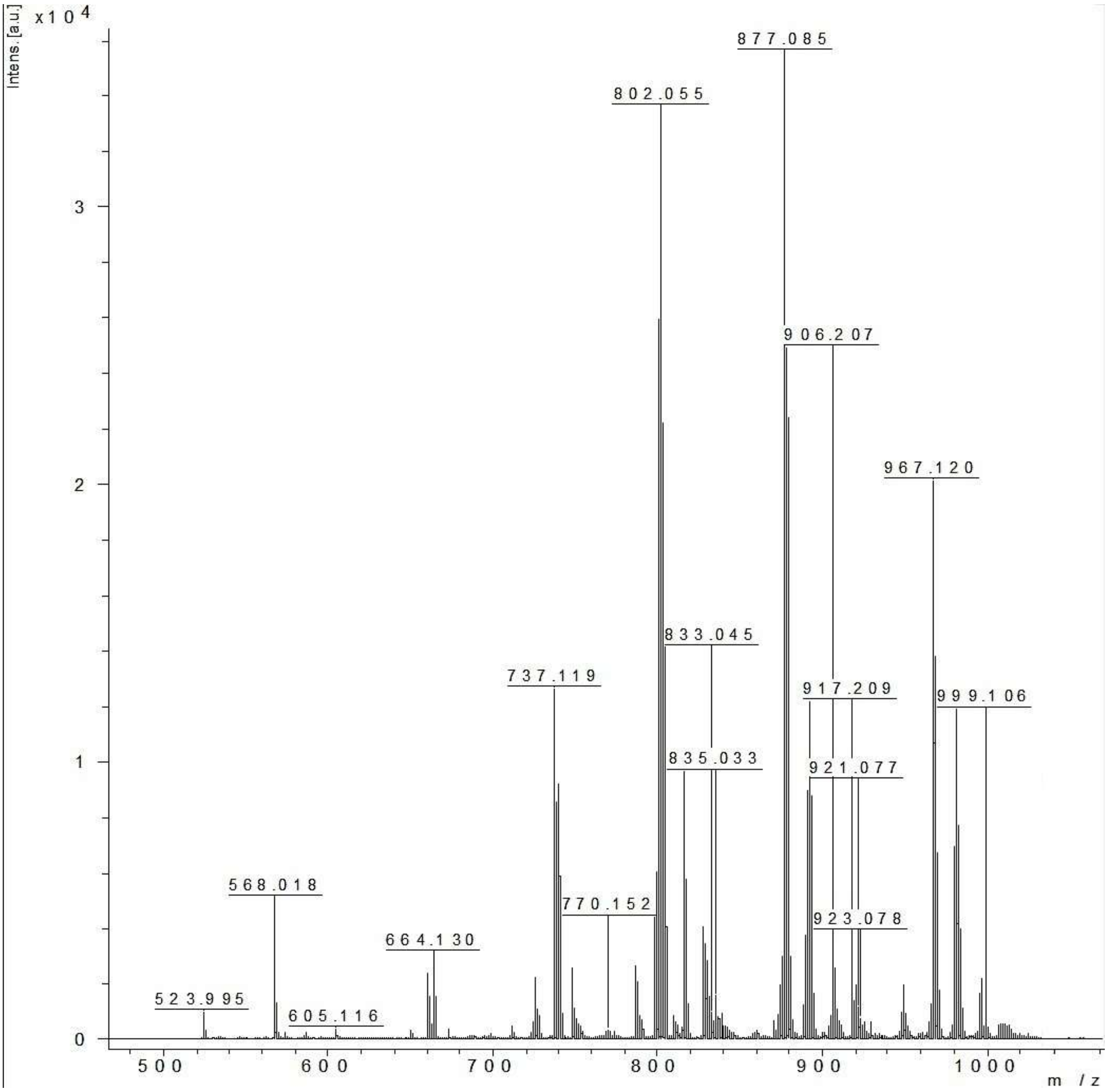

Figure S1a: MALDI spectrum of a crude of the last synthetic step of **PETBP** synthesis

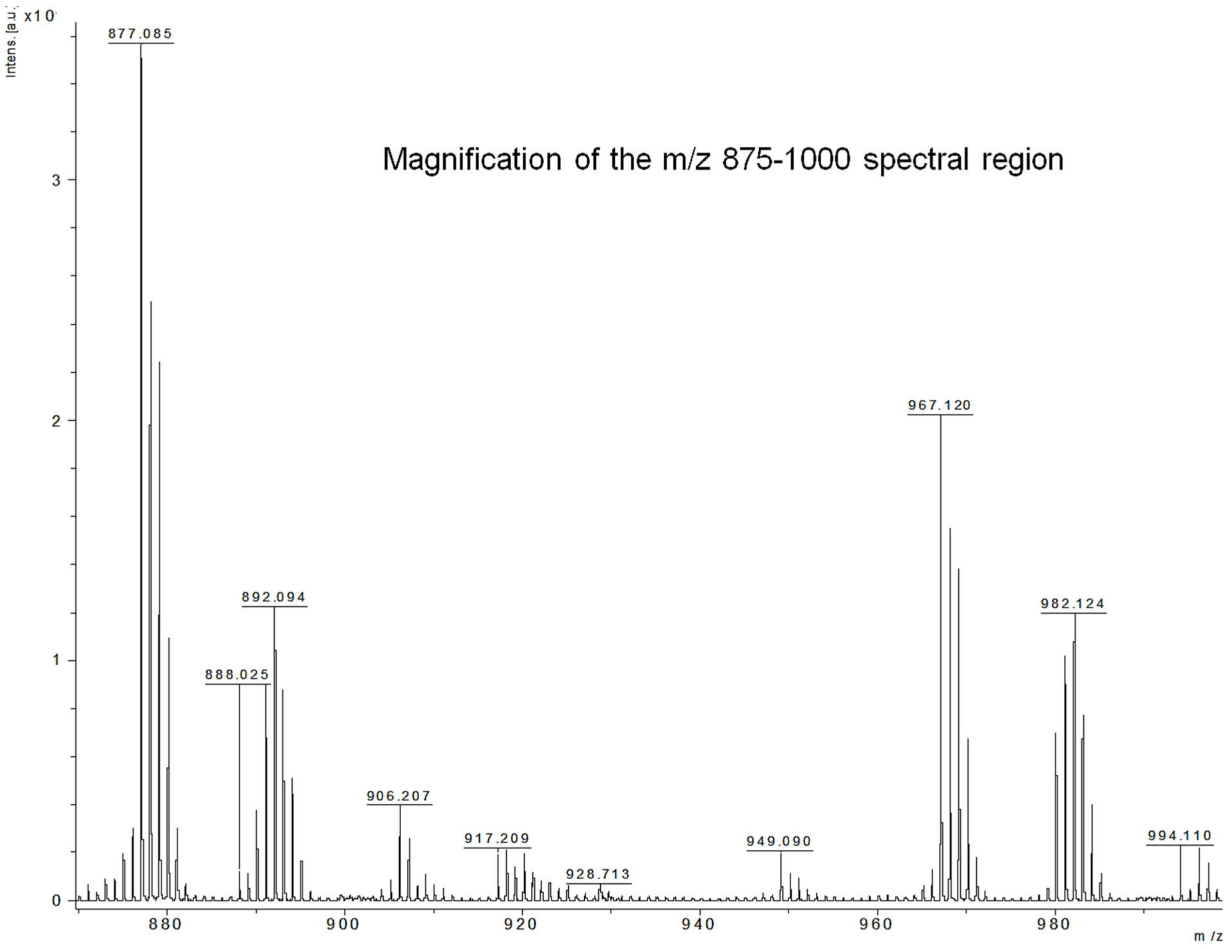

Figure S1b: Magnification of the m/z 870-1000 region

Our MALDI spectra were performed by a third-party analytical laboratory and the results are usually provided from m/z 500 to the highest weighting peak, so we take for granted that there are no chemical species or fragmentations below and above this spectral window, and even below m/z 650 the signals significantly reduce both in number and intensity. Moreover, according to our synthetic path, we assumed that no other carboxylic acid was present in the mixture, granting a selective anchoring of **PETBP** on TiO_2. For this reason, after a chromatographic purification we prepared our solar cells knowing that the photoanode itself would have selectively picked up only our target photosensitizer (figure S2).

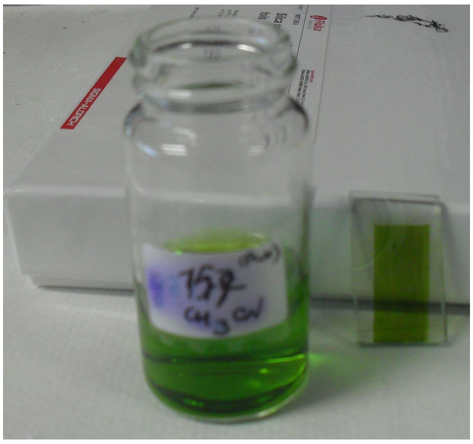

Figure S2: **PETBP**-sensitized TiO_2 film

Concerning the ^{1}H NMR spectrum, shown in figure S3a, we reanalyzed and reassigned the peaks of the aromatic region (figure S3b) but they are not diagnostic to discriminate the nature of the contaminant, because the macrocycle signals are shaped as a multiplet in which only the two benzene-ring signals of the phenylethynyl moiety, diagnostic for our purpose, are distinguishable.

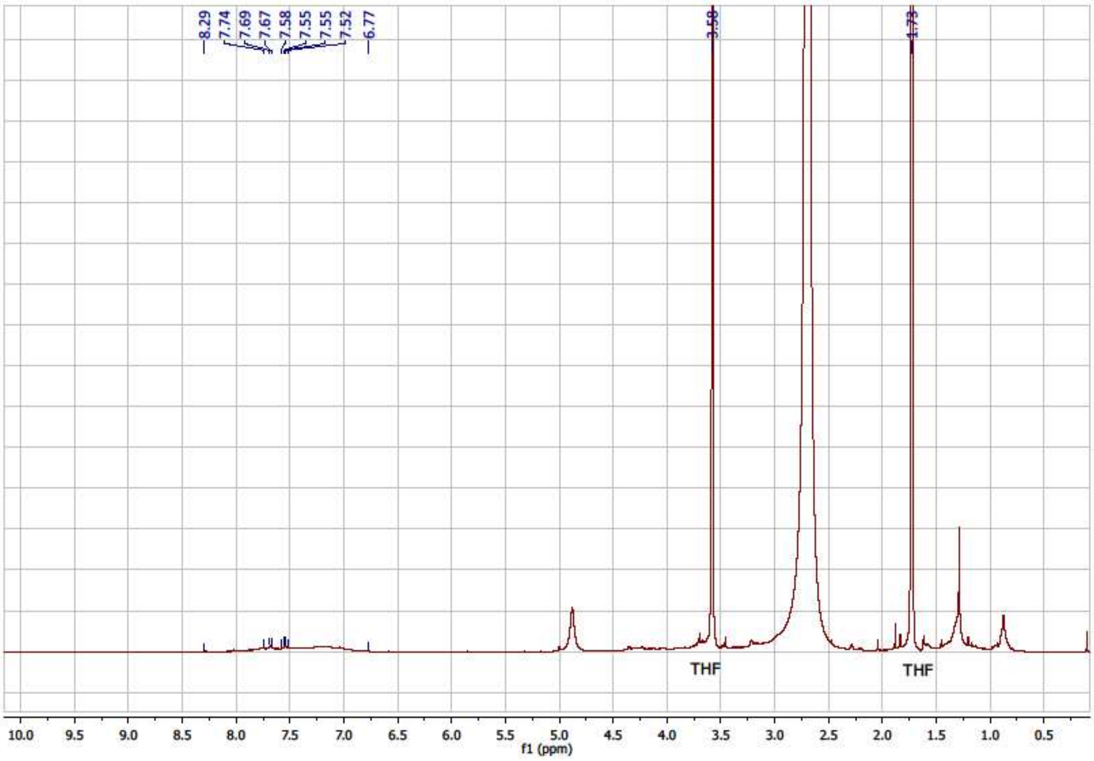

Figure S3a: ^{1}H NMR spectrum of **PETBP**

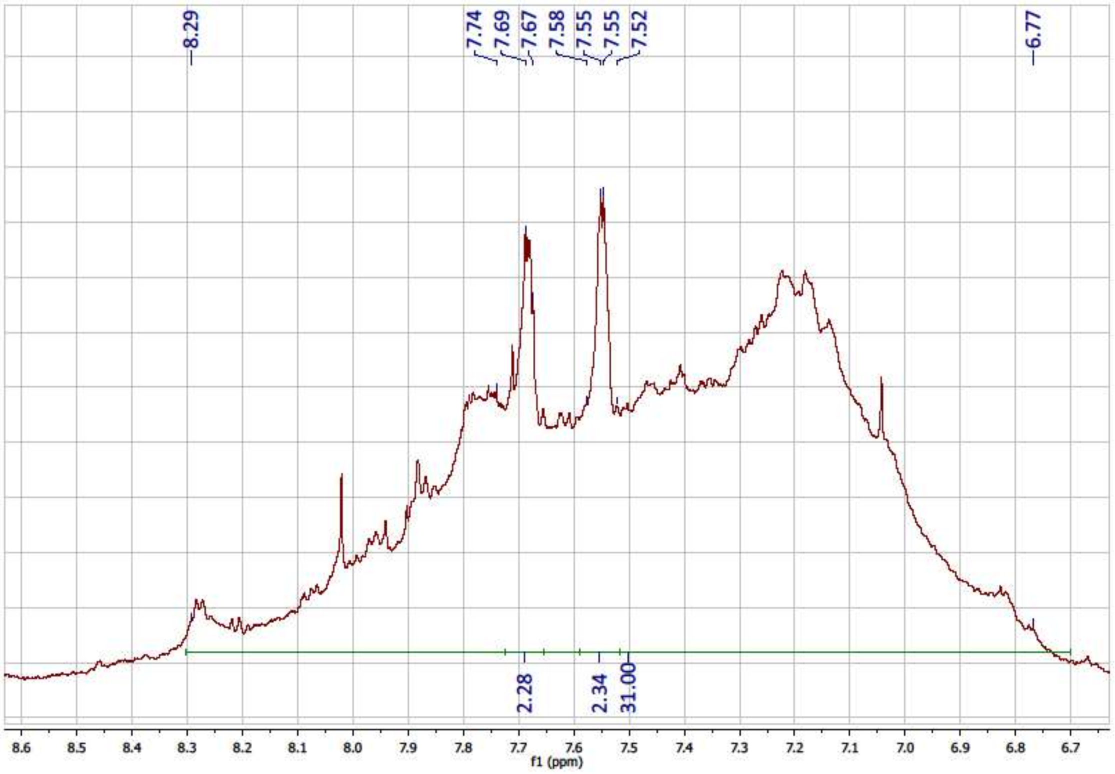

Figure S3b: magnification of the aromatic region of the NMR spectrum

Since the performed integration is self-consistent because there are no other peaks to refer it to, and given the multiplet nature of the signal, it is not possible to distinguish any other diagnostic peak so we cannot exclude that traces of a low-molecular weight aromatic and fluorescent contaminant having a comparable polarity with **PETBP** is present.

A photoluminescent characterization implies a higher sensitivity and extremely diluted samples, so we further purified a small fraction of our mixture by thin layer chromatography, by virtue of the different polarity that our molecule has with respect to any other(s) in the mixture. A UV-Vis analysis confirmed the removal of any macrocyclic impurity: the expected major contaminants (unsubstituted tri-or tetra-phenyltetrabenzoporphyrins) have a sufficiently different absorption behavior on both their main bands to be distinguished, if present.

We are well aware that UV-Vis spectroscopy is not the technique of choice for a rigorous impurity detection, but in this case it was the only feasible characterization due to the low amount of product isolated by TLC. Furthermore, the fluorescence measurements double-checked the absence of macrocyclic contaminants, as we had no evidence of their emission in the investigated regions of the spectrum. For all these reasons we neglected to evaluate if blue region absorbers or low molecular weight contaminants were present, which would not have altered the behavior of **PETBP** as sensitizer in a solar cell.

To summarize, our mistake has been due to the search of a specific class of contaminants, underestimating the possibility to have a low molecular weight

impurity but in this specific case we could not highlight its presence with the performed characterizations.
After having reanalyzed our experimental data we speculated about the chemical nature of the contaminant screening several plausible structures with the assistance of ab initio simulations, choosing (3-oxoisoindolenyl)(3oxoisoindolinydene) as a suitable candidate. We consider it a plausible hypothesis, being reported as a synthetic intermediate and direct precursor of meso-trans-diphenyldi(2-quinolyl)tetrabenzoporphine [Galanin et al, Russian Journal of General Chemistry **2006**, *76*, 148]
